# Personal experience with whole-body, low-dosage, digital X-ray scanning (LODOX-Statscan) in trauma

**DOI:** 10.1186/1757-7241-17-41

**Published:** 2009-09-12

**Authors:** Dimitrios S Evangelopoulos, Simone Deyle, Heinz Zimmermann, Aristomenis K Exadaktylos

**Affiliations:** 1 Department of Emergency Medicine, University Hospital Bern, Switzerland

## Abstract

**Background:**

Lodox-Statscan is a whole-body, skeletal and soft-tissue, low-dose X-ray scanner Anterior-posterior and lateral thoraco-abdominal studies are obtained in 3-5 minutes with only about one-third of the radiation required for conventional radiography. Since its approval by the Food and Drug Administration (FDA) in the USA, several trauma centers have incorporated this technology into their Advanced Trauma Life Support protocols. This review provides a brief overview of the system, and describes the authors' own experience with the system.

**Methods:**

We performed a PubMed search to retrieve all references with 'Lodox' and 'Stat-scan' used as search terms. We furthermore used the google search engine to identify existing alternatives. To the best of our knowledge, this is the only FDA-approved device of its kind currently used in trauma.

**Results and Conclusion:**

The intention of our review has been to sensitize the readership that such alternative devices exist. The key message is that low dosage full body radiography may be an alternative to conventional resuscitation room radiography which is usually a prelude to CT scanning (ATLS algorithm). The combination of both is radiation intensive and therefore we consider any reduction of radiation a success. But only the future will show whether LS will survive in the face of low-dose radiation CT scanners and magnetic resonance imaging devices that may eventually completely replace conventional radiography.

## Introduction

**Figure 1:**
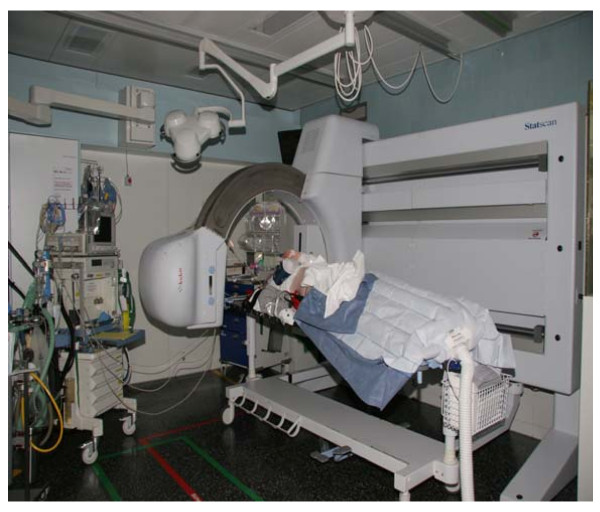
**Lodox Statscan device in Inselspital ED**.

The Lodox-Statscan device (LS) was originally developed for the South African diamond-mining industry to perform low-dose, whole-body scans on mining workers. It has been almost ten years since the LS was first used for medical applications, as reported on by Beningfield in 1999 [1]. The device was approved by the Food and Drug Adminsitration (FDA) in the USA in 2002 for the radiographic examination of both trauma patients and standard emergency patients (Fig. 1). About 25 trauma centers worldwide have now incorporated this technology into their emergency management protocols [2].

LS has also emerged as a useful diagnostic tool in other areas of medicine: recent publications have reported on the effective use of the device in pediatric trauma, pediatrics, neurosurgery, internal medicine, and even in forensic medicine. To the best of our knowledge, this is the only FDA-approved device of its kind currently used in emergency departments. A new full body low dosage 2D/3D scanner (EOS ) has been recently introduced to the international market, but does not seem to be applicable in injured patients.

We hereby present our very personal experience with LS and give an overview on the existing literature and on our own research.

## System description

The LS has an X-ray tube mounted on one end of a C-arm which emits a low-dose, collimated fan-beam of X-rays. The X-ray detector unit is attached to the opposite end of the C-arm and consists of scintillator arrays optically linked to charge-coupled devices [2]. The C-arm travels along the table length at up to 138 mm/s, and a whole-body anterior-posterior (a.p.) scan, takes 13 seconds. The C-arm can be rotated axially around the patient to any angle up to 90°. If desired, subsequent whole-body, horizontal beam, shoot-through lateral, erect, and oblique views can be taken. The unit includes an integrated docking resuscitation table to eliminate transfer from and to trolley and allow complete patient access for ongoing resuscitation. The whole-body images, which can be enlarged for better viewing, are immediately available via a conventional personal computer and PACS. The digital radiation dose relative to the conventional dose varies from 72% (chest) to 2% (pelvis), with a simple average of 6% [3-5]. The radiation skin-entry dose averages 36 mrem (range 18-67), compared with a conventional dose of 591 mrem (range 20-2280) [6]. Effective doses are between 9% and 75% of the United Nations Scientific Committee Report on the Effects of Ionizing Radiation Doses for Standard Examinations [7].

The acquisition costs for the LS are similar to those for conventional hospital imaging products, and material and running costs are low, because the device operates with compatible digital computerized software using conventional computer hardware. In our institution, no additional staff costs, extra staff time or service costs are required, due to the compatibility of system's software.

## Fields of application

### A. Trauma management

#### A. 1. Adult trauma

**Figure 2:**
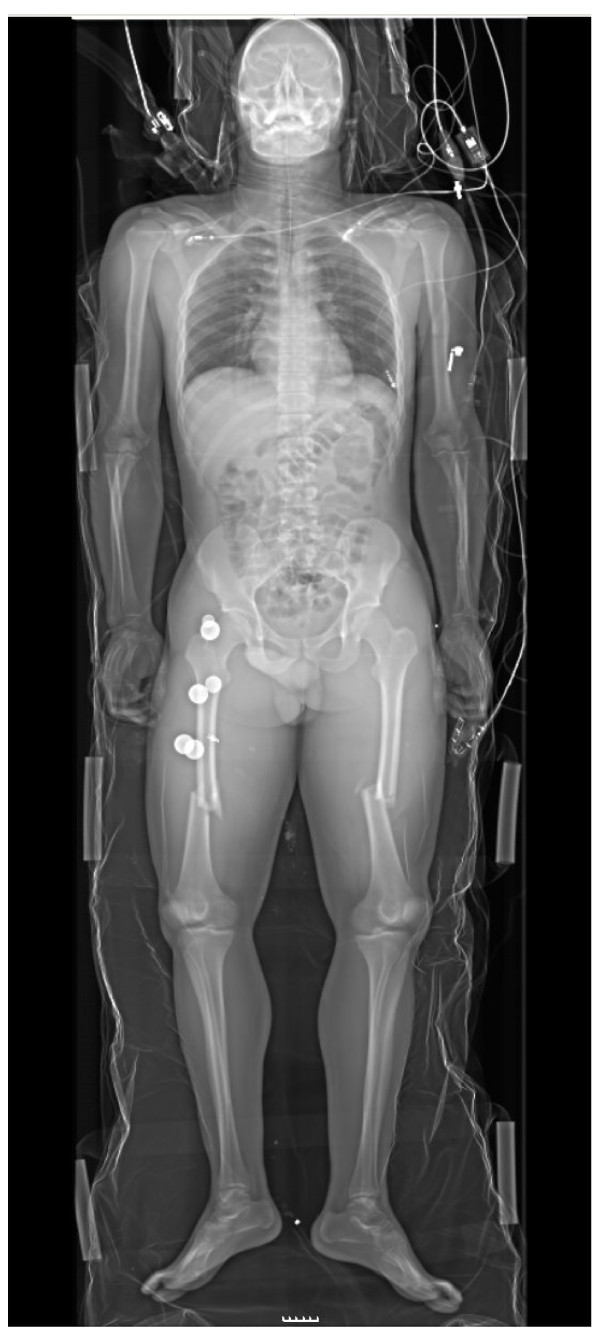
**Whole-body scan of a trauma patient with bilateral femur fractures**.

**Figure 3:**
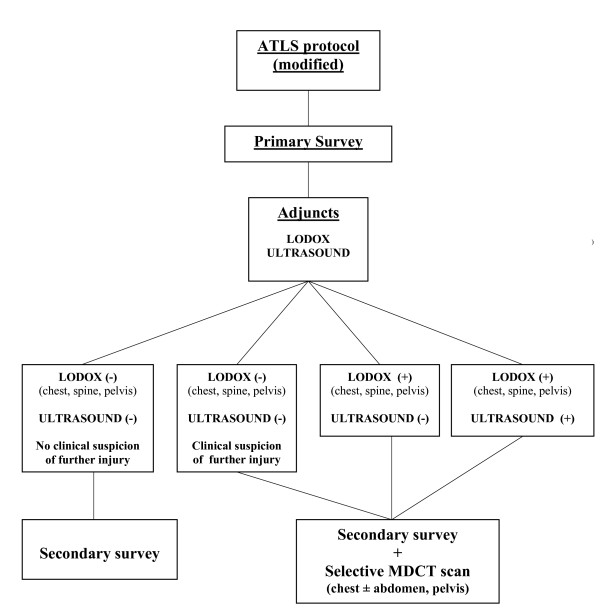
**Bernese Modified ATLS protocol**.

In a clinical trial with the LS, Boffard et al compared its effectiveness in detecting injuries with the standard ATLS X-ray protocol [8]. Compared to conventional a.p. radiographs, the authors reported no loss of information for the chest and pelvis, cervical spine, the cervicothoracic junction, or for long bones (Fig. 2). A study by Beningfield et al of 39 patients compared LS images with conventional images using a scoring system [3]. Although the diagnostic yield of both types of image was similar for most anatomical areas, the digital images were judged superior for the mediastinum, lung, and soft tissues. In a prospective study comparing five-view conventional cervical spine radiographs with the gold standard computed tomography (CT) scan, Shenarts et al detected 54% sensitivity for the conventional X-rays and 96% for the CT scans [9]. In similar studies, Berry et al reported 73% sensitivity, 100% specificity, and a 92% negative predictive value for conventional radiographs in detecting thoracolumbar lesions, and Guillamondegui et al detected an overall sensitivity of 68% and specificity of 98% for conventional pelvic radiographs [10,11]. In a retrospective study, our group assessed the sensitivity and specificity of the LS and CT in injuries of the chest, thoracic spine, lumbar spine, and pelvis [12]. The overall sensitivity of LS imaging was 62%, and specificity was 99%. The sensitivity and specificity findings for individual body regions were similar or even better to those with conventional radiographs. Ethical reasons prevent studies comparing the LS with conventional radiography in our institution in the same patient. In 2008, our group proposed LS as a replacement for the time-consuming basic ATLS X-ray protocol (cervical spine lateral, chest a.p., pelvis a.p.) with a single, rapid, whole-body, a.p. and lateral scan. [13] (Fig. 3). Our aim with the Bernese modified ATLS protocol was to reduce radiography time before starting the secondary survey [14]. We reported a reduction in mean radiography time (from 37 to 26 min). We noted a shorter median whole-body scanning time of 4 min (range: 3-6) with the LS compared to 26 min (8-48) for conventional radiographs [13]. The total emergency room (ER) time, however, was unchanged at a median of 29 min (13-58) compared to 29 min (15-65) with conventional radiographs [13].

#### 2. Pediatric trauma

Radiation has major effects in children. It may affect sensitive developing tissues predisposing to malignant change in later life. Since the risk of cancer induction increases with the radiation dose of each examination, limiting ionizing radiation to minimal levels is the key target for all radiographic imaging protocols in children. Radiation also affects the immature skeleton by interfering with chondrogenesis and reabsorption of calcified cartilage and bone at the growth plate [15].

The low levels of relative digital radiation and radiation skin entry dose with LS imaging compared to conventional radiological doses in adults led to the assumption that it may be a suitable first-choice diagnostic tool for pediatric polytrauma patients [6]. In 2007, Maree et al measured the entry and effective doses of different radiological examinations in children using LS and Shimadzu radiography units [16]. The authors calculated a standard deviation for the entry dose of 0-0.6%. In general, the mean effective dose of the LS was well below that of the Shimadzu unit, and also of those reported in other pediatric radiology studies. For chest examinations, however, the radiation doses with the LS and Shimadzu unit were similar to those in other studies due to the use of chest a.p. projection. Pitcher et al evaluated the role of the LS in pediatric polytrauma and concluded that it was effective for triage, with similar image quality to that of a conventional radiography [17].This led them to revise their polytrauma imaging protocol from the standard ATLS X-rays plus local radiographs if needed to a new protocol comprising an LS a.p. and lateral bodygram. Koning et al and Douglas et al reported shorter imaging times and an enhanced diagnostic yield in the ER [18,19].

### B. Other applications

The LS device has potential too in this field. Beningfield et al reported that the LS radiation dose for proper skull visualization accounted for 16.5% of that of a conventional radiograph [3]. Our group [20] has recently reported on the use of LS for the diagnosis of acute ventriculoperitoneal shunt dysfunction, which we do consider an emergency. Traditionally, the diagnostic protocol in such cases requires serial two-dimensional conventional radiographs of the skull and chest, and also possibly the abdomen, to properly visualize the path of the catheter. Since ventriculoperitoneal shunt malfunction can be a common complication, repeated exposure to radiation may lead to an increased risk of malignancies, but the LS permits a single a.p. bodygram for this procedure with minimal radiation exposure.

Studies from South Africa with a high number of penetrating trauma and a high workload for forensic physicians have shown a benefit of LS in this field [8].

## Personal remarks, summary and outlook

The LS is an FDA-approved new diagnostic tool in emergency medicine. It offers rapid, accurate, whole-body scans in different planes. The availability of whole-body images of injured patients is in our eyes an advantage in better understanding the patients' injury patterns. In our ER, LS has been shown to be equal to or better than conventional radiographs [10,11]. Although CT scanning in ED's remains the gold standard in trauma imaging, its uncritical use has led to increased costs and radiation exposure [21]. The combination of whole-body radiography devices such as LS, focused abdominal sonography for trauma, and a thorough clinical examination may reduce the number of CT scans.

Despite of the fact that the LS has been shown to be effective in excluding thoracic and lumbar spinal trauma, it was less effective in excluding lesions of the cervical spine, which are better visualized by CT. LS is not a CT scanner, and should not be considered as a replacement.

LS scanning can probably look forward to a wide spectrum of new clinical indications in the future because it offers high-speed, high-quality, low-dose, whole-body images in a single scan combined with three-dimensional reconstructive functionality. But only the future will show whether LS will survive in the face of low-dose radiation CT scanners and magnetic resonance imaging devices that may eventually completely replace conventional radiography [22,23].

## List of abbreviations

LS: Lodox-Statscan; FDA: Food and Drug Administration; a.p.: anterior-posterior; ATLS: Advanced Trauma Life Support; CT: computed tomography; ER: emergency room.

## Competing interests

The authors declare that they have no competing interests. The authors exclude any conflict of interest. The paper was not sponsored by Lodox Inc., nor did any of the authors receive financial support for writing the manuscript.

## Authors' contributions

EDS, SD, HZ, AKE: literature search and critical appraisal. EDS, SD, AKE: writing of the manuscript. All authors read and approved the final manuscript.
